# Crystal structures of di­aquadi-μ-hydroxido-tris­[tri­methyl­tin(IV)] diformatotri­methyl­stannate(IV) and di-μ-hydroxido-tris­[tri­methyl­tin(IV)] chloride monohydrate

**DOI:** 10.1107/S2056989016014912

**Published:** 2016-09-30

**Authors:** Felix Otte, Stephan G. Koller, Christopher Golz, Carsten Strohmann

**Affiliations:** a Technische Universität Dortmund, Anorganische Chemie, Otto-Hahn-Strasse 6, D-44227 Dortmund, Germany

**Keywords:** crystal structure, tin, tri­methyl­tin hydroxide, formate, chloride, hydrolysis, hydrogen bonding

## Abstract

The title compounds, [Sn_3_(CH_3_)_9_(OH)_2_(H_2_O)_2_][Sn(CH_3_)_3_(CHO_2_)_2_] (**1**) and [Sn_3_(CH_3_)_9_(OH)_2_]Cl·H_2_O (**2**), are partially condensed products of hydrolysed tri­methyl­tin chloride. In the structures of **1** and **2**, short cationic tris­tannatoxanes (C_9_H_29_O_2_Sn_3_) are bridged by a diformatotri­methyl­tin anion or a chloride anion, respectively. Hydrogen bridges are present and supposedly stabilize these structures against further polymerization to the known polymeric tri­methyl­tin hydroxide. Especially noteworthy is that the formate present in this structure was formed from atmospheric CO_2_.

## Abstract 2

The title compounds are partially condensed products of hydrolysed tri­methyl­tin chloride. In the two structures, short cationic tris­tannatoxanes (C_9_H_29_O_2_Sn_3_) are bridged by a diformatotri­methyl­tin anion or a chloride anion.

## Chemical context   

**Figure 1:**
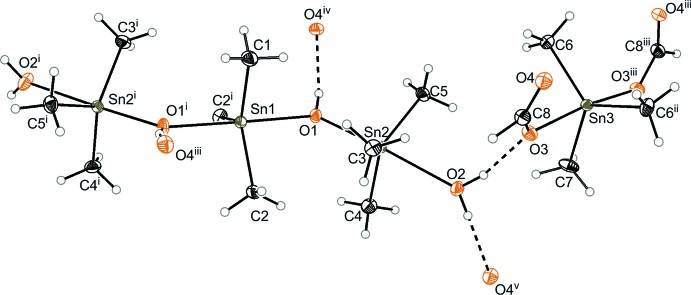
The mol­ecular structure and atom numbering for compound **1**, with displacement ellipsoids drawn at the 30% probability level. [Symmetry codes: (i) 1 − *x*, 2 − *y*, *z*; (ii) 1 − *x*, 1 *- y*, *z*; (iii) 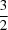 − *x*, 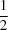 + *y*, −*z*; (iv) −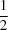 + *x*, 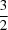 − *y*, −*z;* (v) *x*, *y*, 1 + *z*.]

**Figure 2:**
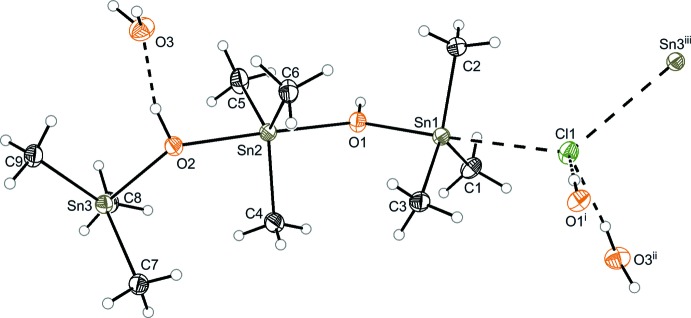
The mol­ecular structure and atom numbering for compound **2**, with displacement ellipsoids drawn at the 30% probability level. [Symmetry codes: (i) 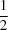 + *x*, −*y*, *z*; (ii) 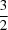 − *x*, *y*, 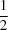 + *z*; (iii) 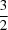 − *x*, −1 + *y*, 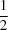 + *z*.]

Nowadays, there are many discussions about climate change and CO_2_ emissions. Therefore, the activation of CO_2_ plays an important role in today’s research. It is already known that CO_2_ is activated by electroreduction of different metals (Machunda *et al.*, 2011 ▸). A selective method to transform CO_2_ into formate uses nanostructured tin catalysts (Zhang *et al.*, 2014 ▸). Compound **1** (Fig. 1 ▸) was formed from atmospheric CO_2_ and thus can be regarded in the context of tin-mediated CO_2_ activation. Compound **2** (Fig. 2 ▸) shows structural analogies and is also discussed herein. Structures **1** and **2** were obtained as byproducts from trapping reactions with tri­methyl­tin chloride (Däschlein *et al.*, 2010 ▸; Unkelbach *et al.*, 2012 ▸; Koller *et al.*, 2015 ▸). 
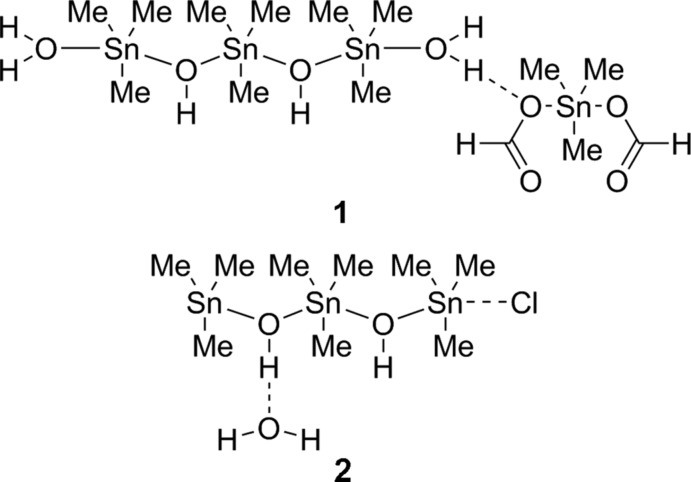


## Structural commentary   

In the crystal structures, no polymeric Sn–O structures were formed, as found in the tri­methyl­tin hydroxide. The short tri­methyl­tin hydroxide chain has a positive and the chloride or bisformatostannate a negative charge. In the structure of **1**, both the cation and the anion are located about a twofold rotation axis whereas in that of **2** all atoms are on general positions. Owing to the presence of hydrogen bonds, there is a change to a smaller Sn—O—Sn angle relative to the polymeric tri­methyl­tin hydroxide (Sn—O—Sn = 140°; Anderson *et al.*, 2011 ▸). In **1**, the Sn1—O1—Sn2 angle is 135.44 (9)° while in **2** it is 135.30 (17)°. In the chloride structure **2**, a change in two further angles is noticed. The O1—Sn1—Cl1 angle [177.58 (10)°] and the O2—Sn3—Cl1′ angle [175.5 (12)°] decreases (compare Lerner *et al.*, 2005 ▸). The water mol­ecules exist in different situations in the two structures. In the formate structure **1**, a water mol­ecule coordinates directly to the Sn2 atom. In compound **2**, the water is embedded in a hydrogen-bonded network between the negatively charged hydroxyl unit (O3⋯H2–O2) and the chloride anion.

## Supra­molecular features   

**Figure 3:**
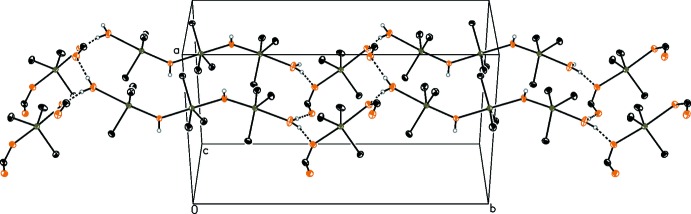
Crystal packing of compound **1**. H atoms not involved in hydrogen bonds have been omitted for clarity. Hydrogen bonds are drawn as black dashed lines (see Table 1 ▸).

As described, both structures are inter­molecularly linked *via* hydrogen bonds. In structure **1** (Fig. 3 ▸ and Table 1 ▸), the formate anion is sterically too demanding to coordinate directly to the outer tin atom of the cationic chain. Therefore, the formate bridges four cationic tris­tannoxanes *via* hydrogen-bonding inter­actions (O3⋯H2*A-*–O2, O4⋯H2*B-*–O2), thus forming a two-dimensional network. Additionally, hydrogen bonds between these sheets form a two-dimensional network along the *bc* plane (O4⋯H1—O1).

**Figure 4:**
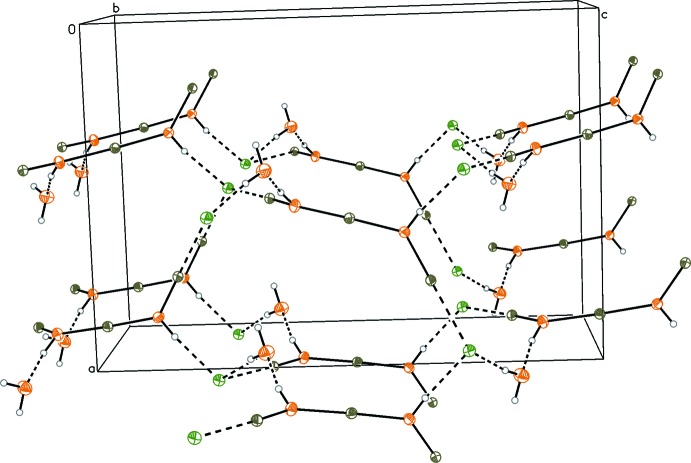
Crystal packing of compound **2**. H atoms not involved in hydrogen bonds have been omitted for clarity. Hydrogen bonds are drawn as black dashed lines (see Table 2 ▸).

In the chloride structure **2** (Fig. 4 ▸ and Table 2 ▸), the chloride anion bridges three cationic tris­tannoxanes, two by Sn⋯Cl inter­actions [Sn1⋯Cl1 = 3.024 (14); Sn3^iii^⋯Cl1 = 3.166 (15) Å], one by a Cl1⋯H1^i^—O1^i^ hydrogen bond [3.251 (4) Å]. A fourth hydrogen bond, Cl1⋯H3^ii^—O3^ii^ [3.068 (5) Å], results in a distorted tetra­hedral environment. Thus, a three-dimensional network of hydrogen bridges is formed. The inter­actions between Sn–Cl differ due to steric repulsion of the C2 and C7^iii^ methyl groups. The van der Waals radius of a methyl group is 2 Å (Brown *et al.*, 2009 ▸) and the distance between the two units is *ca* 3.9 Å.

## Database survey   

The basic building block, tri­methyl­tin hydroxide, has been known for a long time and has been completely characterized (Kraus & Bullard, 1929 ▸; Okawara & Yasuda, 1964 ▸). Since then, studies using single crystal X-ray analysis have been made for the exact structure. A polymeric structure with eight units has been found, which has an angle of *ca* 140° for the Sn—O—Sn bond (Anderson *et al.*, 2011 ▸). Tiekink (1986 ▸) succeeded in obtaining a bis­(tri­methyl­tin)carbonate, wherein the basic polymeric structure has been changed. Here, the tri­methyl­tin units are linked *via* a carbonate. A dimeric structure including chloride as anion and water is also noted. The tin atoms are coordinated by the bridging Cl and HO substituents and angles of 133.2 (2)° for Sn1—Cl1—Sn2 and 179.2 (2)° for O1—Sn1—Cl1 were observed (Lerner *et al.*, 2005 ▸).

## Synthesis and crystallization   

The two structures were obtained as byproducts from trapping reactions with tri­methyl­tin chloride (Strohmann *et al.*, 2006 ▸; Ott *et al.*, 2008 ▸). The samples were stored under atmospheric conditions for a few months. By reaction with atmospheric moisture, partial hydrolysis occurred. In the case of compound **1**, CO_2_ was also activated by a tin-mediated reaction.

## Refinement   

Crystal data, data collection and structure refinement details are summarized in Table 3 ▸. H atoms involved in hydrogen bonding were located in a difference Fourier synthesis map and freely refined. All other H atoms were positioned geometrically and refined using a riding model: C—H = 0.98 Å with *U*_iso_(H) = 1.5*U*_eq_(Cmeth­yl). The CH_3_ hydrogen atoms were allowed to rotate but not to tip. Due to point group symmetry 2 of both the cation and anion in **1**, with the twofold rotation axis running through the respective central Sn atom and one of the methyl groups, the latter is equally disordered over two positions.

## Supplementary Material

Crystal structure: contains datablock(s) Global, 1, 2. DOI: 10.1107/S2056989016014912/su5326sup1.cif

Structure factors: contains datablock(s) 1. DOI: 10.1107/S2056989016014912/su53261sup2.hkl

Structure factors: contains datablock(s) 2. DOI: 10.1107/S2056989016014912/su53262sup3.hkl

CCDC references: 1505529, 1505528

Additional supporting information:  crystallographic information; 3D view; checkCIF report

## Figures and Tables

**Table 1:** Hydrogen-bond geometry (Å, °) for **1** [Chem scheme1]

*D*—H⋯*A*	*D*—H	H⋯*A*	*D*⋯*A*	*D*—H⋯*A*
O2—H2*A*⋯O3	0.87 (2)	1.92 (3)	2.770 (3)	164 (4)
O2—H2*B*⋯O4^i^	0.86 (2)	1.93 (2)	2.791 (3)	178 (3)
O1—H1⋯O4^ii^	0.79 (4)	2.14 (4)	2.917 (3)	167 (3)

Symmetry codes: (i) 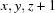; (ii) 
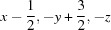
.

**Table 2:** Hydrogen-bond geometry (Å, °) for **2** [Chem scheme1]

*D*—H⋯*A*	*D*—H	H⋯*A*	*D*⋯*A*	*D*—H⋯*A*
O2—H2⋯O3	0.94 (3)	1.81 (3)	2.726 (5)	164 (6)
O1—H1⋯Cl1^i^	0.95 (3)	2.32 (3)	3.251 (4)	168 (6)
O3—H3*D*⋯Cl1^ii^	0.97 (3)	2.10 (3)	3.068 (5)	171 (8)

Symmetry codes: (i) 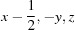; (ii) 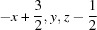.

**Table 3:** Experimental details

	**1**	**2**
Crystal data		
Chemical formula	[Sn_3_(CH_3_)_9_(OH)_2_(H_2_O)_2_][Sn(CH_3_)_3_(CHO_2_)_2_]	[Sn_3_(CH_3_)_9_(OH)_2_]Cl·H_2_O
*M*_r_	407.62	578.86
Crystal system, space group	Orthorhombic, *P*2_1_2_1_2	Orthorhombic, *P* *c* *a*2_1_
Temperature (K)	154	100
*a*, *b*, *c* (Å)	11.0786 (8), 18.9529 (14), 6.6990 (5)	12.623 (3), 8.2675 (18), 18.421 (5)
*V* (Å^3^)	1406.60 (18)	1922.4 (8)
*Z*	4	4
Radiation type	Mo *K*α	Mo *K*α
μ (mm^−1^)	3.54	4.00
Crystal size (mm)	0.16 × 0.10 × 0.08	0.16 × 0.14 × 0.07

Data collection		
Diffractometer	Bruker D8 VENTURE area detector	Bruker D8 VENTURE area detector
Absorption correction	Multi-scan (*SADABS*; Bruker, 2014 ▸)	Multi-scan (*SADABS*; Bruker, 2014 ▸)
*T*_min_, *T*_max_	0.016, 0.038	0.010, 0.032
No. of measured, independent and observed [*I* > 2σ(*I*)] reflections	56576, 3966, 3811	16017, 5320, 5072
*R*_int_	0.036	0.019
(sin θ/λ)_max_ (Å^−1^)	0.696	0.697

Refinement		
*R*[*F* ^2^ > 2σ(*F* ^2^)], *wR*(*F* ^2^), *S*	0.014, 0.027, 1.06	0.022, 0.050, 1.06
No. of reflections	3966	5320
No. of parameters	144	170
No. of restraints	2	5
H-atom treatment	H atoms treated by a mixture of independent and constrained refinement	H atoms treated by a mixture of independent and constrained refinement
Δρ_max_, Δρ_min_ (e Å^−3^)	0.37, −0.33	1.01, −0.38
Absolute structure	Flack *x* determined using 1569 quotients [(*I* ^+^)−(*I* ^−^)]/[(*I* ^+^)+(*I* ^−^)] (Parsons *et al.*, 2013 ▸)	Flack *x* determined using 2271 quotients [(*I* ^+^)−(*I* ^−^)]/[(*I* ^+^)+(*I* ^−^)] (Parsons *et al.*, 2013 ▸)
Absolute structure parameter	−0.040 (19)	−0.026 (19)

Computer programs: *APEX3* and *SAINT* (Bruker, 2014 ▸), *SHELXT* (Sheldrick, 2015*a* ▸), *SHELXL2014* (Sheldrick, 2015*b* ▸) and *OLEX2* (Dolomanov *et al.*, 2009 ▸).

## supplementary crystallographic information

### (1) Diaquadi-µ-hydroxido-tris[trimethyltin(IV)] diformatotrimethylstannate(IV) Crystal data

**Table:**

[Sn_3_(CH_3_)_9_(OH)_2_(H_2_O)_2_][Sn(CH_3_)_3_(CHO_2_)_2_]	*D*_x_ = 1.925 Mg m^−^^3^
*M**_r_* = 407.62	Mo *K*α radiation, λ = 0.71073 Å
Orthorhombic, *P*2_1_2_1_2	Cell parameters from 9917 reflections
*a* = 11.0786 (8) Å	θ = 3–60°
*b* = 18.9529 (14) Å	µ = 3.54 mm^−^^1^
*c* = 6.6990 (5) Å	*T* = 154 K
*V* = 1406.60 (18) Å^3^	Block, colourless
*Z* = 4	0.16 × 0.10 × 0.08 mm
*F*(000) = 784	

### (1) Diaquadi-µ-hydroxido-tris[trimethyltin(IV)] diformatotrimethylstannate(IV) Data collection

**Table:**

Bruker D8 VENTURE area detector diffractometer	3966 independent reflections
Radiation source: microfocus sealed X-ray tube, Incoatec Iµs	3811 reflections with *I* > 2σ(*I*)
HELIOS mirror optics monochromator	*R*_int_ = 0.036
Detector resolution: 10.4167 pixels mm^-1^	θ_max_ = 29.6°, θ_min_ = 2.8°
ω and φ scans	*h* = −15→15
Absorption correction: multi-scan (SADABS; Bruker, 2014)	*k* = −26→26
*T*_min_ = 0.016, *T*_max_ = 0.038	*l* = −9→9
56576 measured reflections	

### (1) Diaquadi-µ-hydroxido-tris[trimethyltin(IV)] diformatotrimethylstannate(IV) Refinement

**Table:**

Refinement on *F*^2^	H atoms treated by a mixture of independent and constrained refinement
Least-squares matrix: full	*w* = 1/[σ^2^(*F*_o_^2^) + (0.0094*P*)^2^ + 0.4247*P*] where *P* = (*F*_o_^2^ + 2*F*_c_^2^)/3
*R*[*F*^2^ > 2σ(*F*^2^)] = 0.014	(Δ/σ)_max_ = 0.004
*wR*(*F*^2^) = 0.027	Δρ_max_ = 0.37 e Å^−^^3^
*S* = 1.06	Δρ_min_ = −0.33 e Å^−^^3^
3966 reflections	Extinction correction: SHELXL2014 (Sheldrick, 2015b), Fc^*^=kFc[1+0.001xFc^2^λ^3^/sin(2θ)]^-1/4^
144 parameters	Extinction coefficient: 0.00294 (12)
2 restraints	Absolute structure: Flack *x* determined using 1569 quotients [(*I*^+^)-(*I*^-^)]/[(*I*^+^)+(*I*^-^)] (Parsons *et al.*, 2013)
Primary atom site location: structure-invariant direct methods	Absolute structure parameter: −0.040 (19)
Hydrogen site location: mixed	

### (1) Diaquadi-µ-hydroxido-tris[trimethyltin(IV)] diformatotrimethylstannate(IV) Special details

**Table:**

Geometry. All esds (except the esd in the dihedral angle between two l.s. planes) are estimated using the full covariance matrix. The cell esds are taken into account individually in the estimation of esds in distances, angles and torsion angles; correlations between esds in cell parameters are only used when they are defined by crystal symmetry. An approximate (isotropic) treatment of cell esds is used for estimating esds involving l.s. planes.

### (1) Diaquadi-µ-hydroxido-tris[trimethyltin(IV)] diformatotrimethylstannate(IV) Fractional atomic coordinates and isotropic or equivalent isotropic displacement parameters (Å^2^)

**Table:**

	*x*	*y*	*z*	*U*_iso_*/*U*_eq_	Occ. (<1)
Sn1	0.5000	1.0000	0.39740 (3)	0.01976 (5)	
Sn2	0.51010 (2)	0.78803 (2)	0.42354 (2)	0.01984 (4)	
Sn3	0.5000	0.5000	0.09198 (3)	0.01949 (5)	
O1	0.43169 (16)	0.88858 (8)	0.3940 (3)	0.0275 (4)	
O2	0.6162 (2)	0.67229 (10)	0.4624 (3)	0.0395 (5)	
H2A	0.621 (4)	0.6398 (17)	0.370 (5)	0.070 (12)*	
H2B	0.639 (3)	0.6499 (15)	0.567 (4)	0.047 (9)*	
O3	0.63477 (16)	0.59175 (9)	0.1188 (3)	0.0275 (4)	
O4	0.69708 (18)	0.60086 (10)	−0.1986 (3)	0.0321 (4)	
C1	0.5000	1.0000	0.0790 (5)	0.0341 (7)	
H1A	0.5212	0.9529	0.0302	0.051*	0.5
H1B	0.5592	1.0343	0.0302	0.051*	0.5
H1C	0.4195	1.0128	0.0302	0.051*	0.5
C2	0.6556 (2)	0.96914 (13)	0.5607 (4)	0.0284 (5)	
H2C	0.6378	0.9263	0.6370	0.043*	
H2D	0.6787	1.0071	0.6524	0.043*	
H2E	0.7221	0.9598	0.4679	0.043*	
C3	0.6662 (2)	0.81086 (14)	0.2519 (4)	0.0306 (6)	
H3A	0.6517	0.8531	0.1709	0.046*	
H3B	0.6842	0.7708	0.1643	0.046*	
H3C	0.7347	0.8192	0.3414	0.046*	
C4	0.5122 (3)	0.78733 (14)	0.7404 (3)	0.0326 (5)	
H4A	0.5957	0.7907	0.7878	0.049*	
H4B	0.4762	0.7433	0.7891	0.049*	
H4C	0.4657	0.8276	0.7908	0.049*	
C5	0.3709 (2)	0.72972 (13)	0.2806 (4)	0.0308 (6)	
H5A	0.2923	0.7504	0.3140	0.046*	
H5B	0.3733	0.6806	0.3261	0.046*	
H5C	0.3827	0.7313	0.1357	0.046*	
C6	0.3758 (2)	0.56431 (13)	−0.0639 (4)	0.0318 (6)	
H6A	0.3182	0.5848	0.0307	0.048*	
H6B	0.3323	0.5357	−0.1622	0.048*	
H6C	0.4195	0.6022	−0.1324	0.048*	
C7	0.5000	0.5000	0.4082 (4)	0.0354 (8)	
H7A	0.5631	0.4681	0.4570	0.053*	0.5
H7B	0.4212	0.4840	0.4570	0.053*	0.5
H7C	0.5156	0.5479	0.4570	0.053*	0.5
C8	0.6991 (3)	0.61657 (14)	−0.0214 (4)	0.0293 (6)	
H8	0.754 (3)	0.6574 (18)	0.034 (5)	0.059 (11)*	
H1	0.365 (3)	0.8863 (17)	0.352 (5)	0.051 (11)*	

### (1) Diaquadi-µ-hydroxido-tris[trimethyltin(IV)] diformatotrimethylstannate(IV) Atomic displacement parameters (Å^2^)

**Table:**

	*U*^11^	*U*^22^	*U*^33^	*U*^12^	*U*^13^	*U*^23^
Sn1	0.01811 (9)	0.01947 (9)	0.02172 (9)	0.00005 (9)	0.000	0.000
Sn2	0.02197 (7)	0.01935 (7)	0.01820 (7)	−0.00208 (7)	−0.00006 (8)	0.00073 (5)
Sn3	0.02102 (9)	0.02158 (9)	0.01588 (9)	0.00227 (9)	0.000	0.000
O1	0.0229 (8)	0.0186 (8)	0.0409 (11)	−0.0023 (6)	−0.0077 (9)	0.0001 (8)
O2	0.0672 (15)	0.0270 (10)	0.0243 (10)	0.0151 (10)	−0.0095 (10)	−0.0033 (8)
O3	0.0312 (9)	0.0298 (9)	0.0215 (9)	−0.0063 (7)	0.0027 (7)	−0.0014 (7)
O4	0.0402 (11)	0.0324 (10)	0.0237 (9)	0.0027 (8)	0.0069 (8)	0.0040 (8)
C1	0.0422 (19)	0.0356 (17)	0.0246 (15)	−0.0051 (19)	0.000	0.000
C2	0.0232 (11)	0.0292 (12)	0.0328 (14)	0.0009 (9)	−0.0063 (11)	−0.0031 (11)
C3	0.0274 (13)	0.0346 (14)	0.0298 (14)	−0.0020 (10)	0.0062 (11)	0.0034 (11)
C4	0.0401 (14)	0.0367 (12)	0.0209 (10)	0.0067 (16)	0.0047 (13)	−0.0009 (9)
C5	0.0332 (14)	0.0272 (12)	0.0320 (14)	−0.0078 (10)	−0.0052 (11)	−0.0016 (10)
C6	0.0341 (13)	0.0263 (12)	0.0350 (15)	0.0070 (10)	−0.0090 (13)	−0.0015 (11)
C7	0.0332 (17)	0.056 (2)	0.0170 (14)	−0.014 (2)	0.000	0.000
C8	0.0310 (14)	0.0290 (14)	0.0280 (14)	−0.0054 (11)	0.0015 (10)	0.0024 (10)

### (1) Diaquadi-µ-hydroxido-tris[trimethyltin(IV)] diformatotrimethylstannate(IV) Geometric parameters (Å, º)

**Table:**

Sn1—O1	2.2433 (16)	C1—H1B	0.9800
Sn1—O1^i^	2.2433 (16)	C1—H1C	0.9800
Sn1—C1	2.133 (3)	C2—H2C	0.9800
Sn1—C2	2.124 (2)	C2—H2D	0.9800
Sn1—C2^i^	2.124 (2)	C2—H2E	0.9800
Sn2—O1	2.1036 (16)	C3—H3A	0.9800
Sn2—O2	2.5023 (19)	C3—H3B	0.9800
Sn2—C3	2.121 (2)	C3—H3C	0.9800
Sn2—C4	2.123 (2)	C4—H4A	0.9800
Sn2—C5	2.126 (2)	C4—H4B	0.9800
Sn3—O3^ii^	2.2991 (17)	C4—H4C	0.9800
Sn3—O3	2.2990 (17)	C5—H5A	0.9800
Sn3—C6	2.114 (2)	C5—H5B	0.9800
Sn3—C6^ii^	2.114 (2)	C5—H5C	0.9800
Sn3—C7	2.119 (3)	C6—H6A	0.9800
O1—H1	0.79 (4)	C6—H6B	0.9800
O2—H2A	0.87 (2)	C6—H6C	0.9800
O2—H2B	0.86 (2)	C7—H7A	0.9800
O3—C8	1.269 (3)	C7—H7B	0.9800
O4—C8	1.224 (3)	C7—H7C	0.9800
C1—H1A	0.9800	C8—H8	1.05 (3)
			
O1—Sn1—O1^i^	178.83 (11)	H1A—C1—H1C	109.5
C1—Sn1—O1^i^	89.42 (5)	H1B—C1—H1C	109.5
C1—Sn1—O1	89.42 (5)	Sn1—C2—H2C	109.5
C2—Sn1—O1	91.14 (8)	Sn1—C2—H2D	109.5
C2—Sn1—O1^i^	89.46 (8)	Sn1—C2—H2E	109.5
C2^i^—Sn1—O1	89.46 (8)	H2C—C2—H2D	109.5
C2^i^—Sn1—O1^i^	91.14 (8)	H2C—C2—H2E	109.5
C2^i^—Sn1—C1	121.00 (7)	H2D—C2—H2E	109.5
C2—Sn1—C1	121.00 (7)	Sn2—C3—H3A	109.5
C2—Sn1—C2^i^	118.01 (15)	Sn2—C3—H3B	109.5
O1—Sn2—O2	176.30 (8)	Sn2—C3—H3C	109.5
O1—Sn2—C3	95.79 (9)	H3A—C3—H3B	109.5
O1—Sn2—C4	95.99 (9)	H3A—C3—H3C	109.5
O1—Sn2—C5	97.41 (9)	H3B—C3—H3C	109.5
C3—Sn2—O2	81.51 (9)	Sn2—C4—H4A	109.5
C3—Sn2—C4	122.30 (12)	Sn2—C4—H4B	109.5
C3—Sn2—C5	116.97 (11)	Sn2—C4—H4C	109.5
C4—Sn2—O2	83.41 (9)	H4A—C4—H4B	109.5
C4—Sn2—C5	117.08 (11)	H4A—C4—H4C	109.5
C5—Sn2—O2	86.09 (9)	H4B—C4—H4C	109.5
O3—Sn3—O3^ii^	171.04 (9)	Sn2—C5—H5A	109.5
C6—Sn3—O3^ii^	92.99 (9)	Sn2—C5—H5B	109.5
C6^ii^—Sn3—O3^ii^	91.44 (9)	Sn2—C5—H5C	109.5
C6^ii^—Sn3—O3	92.99 (9)	H5A—C5—H5B	109.5
C6—Sn3—O3	91.43 (9)	H5A—C5—H5C	109.5
C6^ii^—Sn3—C6	120.80 (16)	H5B—C5—H5C	109.5
C6—Sn3—C7	119.60 (8)	Sn3—C6—H6A	109.5
C6^ii^—Sn3—C7	119.60 (8)	Sn3—C6—H6B	109.5
C7—Sn3—O3^ii^	85.52 (4)	Sn3—C6—H6C	109.5
C7—Sn3—O3	85.52 (4)	H6A—C6—H6B	109.5
Sn1—O1—H1	112 (2)	H6A—C6—H6C	109.5
Sn2—O1—Sn1	135.44 (9)	H6B—C6—H6C	109.5
Sn2—O1—H1	112 (2)	Sn3—C7—H7A	109.5
Sn2—O2—H2A	125 (3)	Sn3—C7—H7B	109.5
Sn2—O2—H2B	131 (2)	Sn3—C7—H7C	109.5
H2A—O2—H2B	102 (3)	H7A—C7—H7B	109.5
C8—O3—Sn3	125.94 (17)	H7A—C7—H7C	109.5
Sn1—C1—H1A	109.5	H7B—C7—H7C	109.5
Sn1—C1—H1B	109.5	O3—C8—H8	110 (2)
Sn1—C1—H1C	109.5	O4—C8—O3	128.1 (3)
H1A—C1—H1B	109.5	O4—C8—H8	122 (2)
			
Sn3—O3—C8—O4	5.5 (4)		

Symmetry codes: (i) −*x*+1, −*y*+2, *z*; (ii) −*x*+1, −*y*+1, *z*.

### (1) Diaquadi-µ-hydroxido-tris[trimethyltin(IV)] diformatotrimethylstannate(IV) Hydrogen-bond geometry (Å, º)

**Table:**

*D*—H···*A*	*D*—H	H···*A*	*D*···*A*	*D*—H···*A*
O2—H2*A*···O3	0.87 (2)	1.92 (3)	2.770 (3)	164 (4)
O2—H2*B*···O4^iii^	0.86 (2)	1.93 (2)	2.791 (3)	178 (3)
O1—H1···O4^iv^	0.79 (4)	2.14 (4)	2.917 (3)	167 (3)

Symmetry codes: (iii) *x*, *y*, *z*+1; (iv) *x*−1/2, −*y*+3/2, −*z*.

### (2) Di-µ-hydroxido-tris[trimethyltin(IV)] chloride monohydrate Crystal data

**Table:**

[Sn_3_(CH_3_)_9_(OH)_2_]Cl·H_2_O	*D*_x_ = 2.000 Mg m^−^^3^
*M**_r_* = 578.86	Mo *K*α radiation, λ = 0.71073 Å
Orthorhombic, *P**c**a*2_1_	Cell parameters from 9903 reflections
*a* = 12.623 (3) Å	θ = 2.9–29.6°
*b* = 8.2675 (18) Å	µ = 4.00 mm^−^^1^
*c* = 18.421 (5) Å	*T* = 100 K
*V* = 1922.4 (8) Å^3^	Block, colourless
*Z* = 4	0.16 × 0.14 × 0.07 mm
*F*(000) = 1104	

### (2) Di-µ-hydroxido-tris[trimethyltin(IV)] chloride monohydrate Data collection

**Table:**

Bruker D8 VENTURE area detector diffractometer	5072 reflections with *I* > 2σ(*I*)
Detector resolution: 10.4167 pixels mm^-1^	*R*_int_ = 0.019
φ and ω scans	θ_max_ = 29.7°, θ_min_ = 2.2°
Absorption correction: multi-scan (SADABS; Bruker, 2014)	*h* = −15→17
*T*_min_ = 0.010, *T*_max_ = 0.032	*k* = −11→11
16017 measured reflections	*l* = −25→25
5320 independent reflections	

### (2) Di-µ-hydroxido-tris[trimethyltin(IV)] chloride monohydrate Refinement

**Table:**

Refinement on *F*^2^	Hydrogen site location: mixed
Least-squares matrix: full	H atoms treated by a mixture of independent and constrained refinement
*R*[*F*^2^ > 2σ(*F*^2^)] = 0.022	*w* = 1/[σ^2^(*F*_o_^2^) + (0.0269*P*)^2^ + 0.6817*P*] where *P* = (*F*_o_^2^ + 2*F*_c_^2^)/3
*wR*(*F*^2^) = 0.050	(Δ/σ)_max_ = 0.001
*S* = 1.06	Δρ_max_ = 1.01 e Å^−^^3^
5320 reflections	Δρ_min_ = −0.38 e Å^−^^3^
170 parameters	Absolute structure: Flack *x* determined using 2271 quotients [(*I*^+^)-(*I*^-^)]/[(*I*^+^)+(*I*^-^)] (Parsons *et al.*, 2013)
5 restraints	Absolute structure parameter: −0.026 (19)
Primary atom site location: structure-invariant direct methods	

### (2) Di-µ-hydroxido-tris[trimethyltin(IV)] chloride monohydrate Special details

**Table:**

Geometry. All esds (except the esd in the dihedral angle between two l.s. planes) are estimated using the full covariance matrix. The cell esds are taken into account individually in the estimation of esds in distances, angles and torsion angles; correlations between esds in cell parameters are only used when they are defined by crystal symmetry. An approximate (isotropic) treatment of cell esds is used for estimating esds involving l.s. planes.

### (2) Di-µ-hydroxido-tris[trimethyltin(IV)] chloride monohydrate Fractional atomic coordinates and isotropic or equivalent isotropic displacement parameters (Å^2^)

**Table:**

	*x*	*y*	*z*	*U*_iso_*/*U*_eq_	
C1	0.7513 (5)	0.1853 (7)	0.7655 (3)	0.0397 (11)	
H1A	0.7352	0.2995	0.7566	0.059*	
H1B	0.8181	0.1767	0.7924	0.059*	
H1C	0.6941	0.1366	0.7941	0.059*	
C2	0.7054 (4)	−0.1756 (6)	0.6542 (3)	0.0345 (10)	
H2A	0.6448	−0.1902	0.6868	0.052*	
H2B	0.7609	−0.2537	0.6667	0.052*	
H2C	0.6828	−0.1931	0.6039	0.052*	
C3	0.8814 (4)	0.1523 (7)	0.5927 (3)	0.0358 (11)	
H3A	0.8661	0.1136	0.5435	0.054*	
H3B	0.9515	0.1141	0.6077	0.054*	
H3C	0.8802	0.2709	0.5933	0.054*	
C4	0.6887 (5)	0.4752 (7)	0.5271 (3)	0.0436 (13)	
H4A	0.7587	0.4640	0.5046	0.065*	
H4B	0.6969	0.4882	0.5797	0.065*	
H4C	0.6527	0.5702	0.5071	0.065*	
C5	0.4333 (5)	0.2706 (8)	0.5290 (3)	0.0490 (15)	
H5A	0.4053	0.3790	0.5191	0.073*	
H5B	0.4221	0.2438	0.5802	0.073*	
H5C	0.3965	0.1915	0.4984	0.073*	
C6	0.6638 (5)	0.0545 (6)	0.4578 (3)	0.0381 (11)	
H6A	0.6173	0.0158	0.4189	0.057*	
H6B	0.6712	−0.0298	0.4948	0.057*	
H6C	0.7336	0.0803	0.4376	0.057*	
C7	0.7215 (4)	0.6488 (6)	0.3435 (3)	0.0367 (10)	
H7A	0.7630	0.5597	0.3640	0.055*	
H7B	0.7275	0.7439	0.3750	0.055*	
H7C	0.7485	0.6753	0.2950	0.055*	
C8	0.4542 (4)	0.7036 (6)	0.4051 (3)	0.0359 (11)	
H8A	0.3839	0.6535	0.4026	0.054*	
H8B	0.4493	0.8169	0.3898	0.054*	
H8C	0.4806	0.6985	0.4551	0.054*	
C9	0.5063 (5)	0.4823 (7)	0.2352 (3)	0.0390 (11)	
H9A	0.5524	0.5207	0.1960	0.059*	
H9B	0.4335	0.5179	0.2260	0.059*	
H9C	0.5085	0.3639	0.2371	0.059*	
O1	0.6340 (3)	0.1729 (4)	0.61594 (19)	0.0327 (7)	
H1	0.575 (4)	0.168 (9)	0.648 (3)	0.06 (2)*	
O2	0.5652 (3)	0.3638 (4)	0.39350 (19)	0.0333 (7)	
H2	0.519 (4)	0.293 (6)	0.369 (3)	0.040 (16)*	
Sn1	0.76540 (2)	0.06256 (3)	0.66540 (2)	0.02692 (7)	
Sn2	0.59742 (2)	0.26536 (3)	0.50553 (2)	0.02755 (7)	
Sn3	0.55935 (2)	0.57842 (4)	0.33566 (2)	0.02786 (7)	
O3	0.4394 (3)	0.1219 (5)	0.3417 (3)	0.0488 (10)	
H3D	0.467 (7)	0.043 (8)	0.308 (4)	0.08 (3)*	
H3E	0.365 (4)	0.127 (15)	0.328 (10)	0.18 (6)*	
Cl1	0.94986 (10)	−0.10984 (17)	0.73601 (7)	0.0379 (3)	

### (2) Di-µ-hydroxido-tris[trimethyltin(IV)] chloride monohydrate Atomic displacement parameters (Å^2^)

**Table:**

	*U*^11^	*U*^22^	*U*^33^	*U*^12^	*U*^13^	*U*^23^
C1	0.037 (3)	0.049 (3)	0.033 (3)	0.000 (3)	−0.002 (2)	−0.011 (2)
C2	0.033 (2)	0.034 (2)	0.037 (3)	−0.0031 (19)	−0.002 (2)	0.0016 (19)
C3	0.029 (2)	0.042 (3)	0.036 (3)	−0.003 (2)	0.002 (2)	0.004 (2)
C4	0.058 (4)	0.037 (3)	0.035 (3)	−0.009 (3)	−0.009 (2)	0.003 (2)
C5	0.035 (3)	0.068 (4)	0.044 (3)	0.007 (3)	0.004 (2)	0.019 (3)
C6	0.048 (3)	0.034 (2)	0.032 (2)	0.007 (2)	−0.006 (2)	−0.0010 (19)
C7	0.032 (2)	0.044 (3)	0.034 (2)	−0.002 (2)	0.000 (2)	0.006 (2)
C8	0.036 (3)	0.041 (3)	0.031 (2)	0.003 (2)	0.003 (2)	0.002 (2)
C9	0.046 (3)	0.039 (3)	0.032 (2)	0.000 (2)	−0.008 (2)	0.001 (2)
O1	0.0276 (17)	0.0410 (18)	0.0295 (17)	0.0025 (15)	0.0020 (14)	0.0056 (14)
O2	0.041 (2)	0.0288 (17)	0.0306 (17)	−0.0010 (15)	−0.0037 (15)	0.0010 (13)
Sn1	0.02689 (14)	0.02985 (14)	0.02403 (13)	−0.00150 (11)	0.00091 (13)	0.00026 (11)
Sn2	0.02642 (14)	0.02809 (14)	0.02815 (14)	0.00031 (11)	−0.00014 (13)	0.00004 (12)
Sn3	0.02768 (15)	0.02928 (14)	0.02664 (14)	0.00066 (11)	−0.00002 (13)	0.00054 (12)
O3	0.047 (2)	0.045 (2)	0.054 (3)	0.0023 (18)	−0.005 (2)	−0.011 (2)
Cl1	0.0311 (6)	0.0487 (7)	0.0339 (6)	0.0014 (5)	−0.0004 (5)	0.0028 (5)

### (2) Di-µ-hydroxido-tris[trimethyltin(IV)] chloride monohydrate Geometric parameters (Å, º)

**Table:**

C1—H1A	0.9800	C6—Sn2	2.125 (5)
C1—H1B	0.9800	C7—H7A	0.9800
C1—H1C	0.9800	C7—H7B	0.9800
C1—Sn1	2.113 (5)	C7—H7C	0.9800
C2—H2A	0.9800	C7—Sn3	2.133 (5)
C2—H2B	0.9800	C8—H8A	0.9800
C2—H2C	0.9800	C8—H8B	0.9800
C2—Sn1	2.120 (5)	C8—H8C	0.9800
C3—H3A	0.9800	C8—Sn3	2.114 (5)
C3—H3B	0.9800	C9—H9A	0.9800
C3—H3C	0.9800	C9—H9B	0.9800
C3—Sn1	2.118 (5)	C9—H9C	0.9800
C4—H4A	0.9800	C9—Sn3	2.123 (5)
C4—H4B	0.9800	O1—H1	0.95 (3)
C4—H4C	0.9800	O1—Sn1	2.100 (3)
C4—Sn2	2.120 (5)	O1—Sn2	2.222 (3)
C5—H5A	0.9800	O2—H2	0.94 (3)
C5—H5B	0.9800	O2—Sn2	2.255 (4)
C5—H5C	0.9800	O2—Sn3	2.071 (3)
C5—Sn2	2.117 (6)	Sn1—Cl1	3.0240 (14)
C6—H6A	0.9800	O3—H3D	0.97 (3)
C6—H6B	0.9800	O3—H3E	0.98 (3)
C6—H6C	0.9800	Cl1—Sn3^i^	3.1663 (15)
			
H1A—C1—H1B	109.5	H8B—C8—H8C	109.5
H1A—C1—H1C	109.5	Sn3—C8—H8A	109.5
H1B—C1—H1C	109.5	Sn3—C8—H8B	109.5
Sn1—C1—H1A	109.5	Sn3—C8—H8C	109.5
Sn1—C1—H1B	109.5	H9A—C9—H9B	109.5
Sn1—C1—H1C	109.5	H9A—C9—H9C	109.5
H2A—C2—H2B	109.5	H9B—C9—H9C	109.5
H2A—C2—H2C	109.5	Sn3—C9—H9A	109.5
H2B—C2—H2C	109.5	Sn3—C9—H9B	109.5
Sn1—C2—H2A	109.5	Sn3—C9—H9C	109.5
Sn1—C2—H2B	109.5	Sn1—O1—H1	109 (4)
Sn1—C2—H2C	109.5	Sn1—O1—Sn2	135.30 (17)
H3A—C3—H3B	109.5	Sn2—O1—H1	115 (4)
H3A—C3—H3C	109.5	Sn2—O2—H2	109 (4)
H3B—C3—H3C	109.5	Sn3—O2—H2	105 (4)
Sn1—C3—H3A	109.5	Sn3—O2—Sn2	141.84 (17)
Sn1—C3—H3B	109.5	C1—Sn1—C2	120.1 (2)
Sn1—C3—H3C	109.5	C1—Sn1—C3	116.2 (2)
H4A—C4—H4B	109.5	C1—Sn1—Cl1	85.16 (17)
H4A—C4—H4C	109.5	C2—Sn1—Cl1	83.06 (14)
H4B—C4—H4C	109.5	C3—Sn1—C2	120.7 (2)
Sn2—C4—H4A	109.5	C3—Sn1—Cl1	84.55 (15)
Sn2—C4—H4B	109.5	O1—Sn1—C1	95.96 (19)
Sn2—C4—H4C	109.5	O1—Sn1—C2	94.52 (17)
H5A—C5—H5B	109.5	O1—Sn1—C3	96.85 (17)
H5A—C5—H5C	109.5	O1—Sn1—Cl1	177.58 (10)
H5B—C5—H5C	109.5	C4—Sn2—C6	122.3 (3)
Sn2—C5—H5A	109.5	C4—Sn2—O1	89.81 (18)
Sn2—C5—H5B	109.5	C4—Sn2—O2	88.55 (17)
Sn2—C5—H5C	109.5	C5—Sn2—C4	118.5 (3)
H6A—C6—H6B	109.5	C5—Sn2—C6	119.2 (3)
H6A—C6—H6C	109.5	C5—Sn2—O1	91.36 (18)
H6B—C6—H6C	109.5	C5—Sn2—O2	90.15 (19)
Sn2—C6—H6A	109.5	C6—Sn2—O1	90.83 (17)
Sn2—C6—H6B	109.5	C6—Sn2—O2	89.35 (17)
Sn2—C6—H6C	109.5	O1—Sn2—O2	178.17 (14)
H7A—C7—H7B	109.5	C8—Sn3—C7	115.3 (2)
H7A—C7—H7C	109.5	C8—Sn3—C9	120.9 (2)
H7B—C7—H7C	109.5	C9—Sn3—C7	117.6 (2)
Sn3—C7—H7A	109.5	O2—Sn3—C7	99.50 (18)
Sn3—C7—H7B	109.5	O2—Sn3—C8	97.50 (17)
Sn3—C7—H7C	109.5	O2—Sn3—C9	97.99 (18)
H8A—C8—H8B	109.5	H3D—O3—H3E	102 (10)
H8A—C8—H8C	109.5	Sn1—Cl1—Sn3^i^	127.21 (4)

Symmetry code: (i) −*x*+3/2, *y*−1, *z*+1/2.

### (2) Di-µ-hydroxido-tris[trimethyltin(IV)] chloride monohydrate Hydrogen-bond geometry (Å, º)

**Table:**

*D*—H···*A*	*D*—H	H···*A*	*D*···*A*	*D*—H···*A*
O2—H2···O3	0.94 (3)	1.81 (3)	2.726 (5)	164 (6)
O1—H1···Cl1^ii^	0.95 (3)	2.32 (3)	3.251 (4)	168 (6)
O3—H3*D*···Cl1^iii^	0.97 (3)	2.10 (3)	3.068 (5)	171 (8)

Symmetry codes: (ii) *x*−1/2, −*y*, *z*; (iii) −*x*+3/2, *y*, *z*−1/2.
